# An Intelligent ECG-Based Tool for Diagnosing COVID-19 via Ensemble Deep Learning Techniques

**DOI:** 10.3390/bios12050299

**Published:** 2022-05-05

**Authors:** Omneya Attallah

**Affiliations:** Department of Electronics and Communications Engineering, College of Engineering and Technology, Arab Academy for Science, Technology and Maritime Transport, Alexandria 1029, Egypt; o.attallah@aast.edu

**Keywords:** deep learning, COVID-19, ECG trace image, transfer learning, Convolutional Neural Networks (CNN), feature selection

## Abstract

Diagnosing COVID-19 accurately and rapidly is vital to control its quick spread, lessen lockdown restrictions, and decrease the workload on healthcare structures. The present tools to detect COVID-19 experience numerous shortcomings. Therefore, novel diagnostic tools are to be examined to enhance diagnostic accuracy and avoid the limitations of these tools. Earlier studies indicated multiple structures of cardiovascular alterations in COVID-19 cases which motivated the realization of using ECG data as a tool for diagnosing the novel coronavirus. This study introduced a novel automated diagnostic tool based on ECG data to diagnose COVID-19. The introduced tool utilizes ten deep learning (DL) models of various architectures. It obtains significant features from the last fully connected layer of each DL model and then combines them. Afterward, the tool presents a hybrid feature selection based on the chi-square test and sequential search to select significant features. Finally, it employs several machine learning classifiers to perform two classification levels. A binary level to differentiate between normal and COVID-19 cases, and a multiclass to discriminate COVID-19 cases from normal and other cardiac complications. The proposed tool reached an accuracy of 98.2% and 91.6% for binary and multiclass levels, respectively. This performance indicates that the ECG could be used as an alternative means of diagnosis of COVID-19.

## 1. Introduction

At the end of December 2019, the world faced a new type of threatening disease called coronavirus, commonly known as COVID-19 [1]. Based on statistics announced by the World Health Organization (WHO) [2], more than 190 million cases of COVID-19 and more than 4 million cases of mortality have been reported worldwide on 31 July 2021. Due to the rapid propagation and the massive increase in the number of new infections of such a disease, the world faced new challenges [3]. These challenges involved travel constraints, countries’ lockdown, social distancing, and curfews. Most importantly, healthcare associations of many countries were about to collapse due to the superfluous number of COVID-19 infections that needed beds and deficiencies in vital medical kits and supplies. Consequently, the rapid and precise diagnosis of COVID-19 is important to lower mortality rates and avert the encumbrance on health organizations.

Based on the COVID-19 diagnosis provided by the Chinese government, the real-time reverse transcription-polymerase chain reaction (RT-PCR) test is the gold standard for the diagnosis of COVID-19 [4]. However, late sample acquisition, firm laboratory setting restrictions, and the requirement of qualified experts to perform the RT-PCR exam could lead to a prolonged and inaccurate diagnosis [5]. Therefore, more efficient methods are needed to achieve a more precise and faster diagnosis. Among these approaches are antigen tests and medical imaging, including computed tomography (CT) and X-ray imaging techniques. Although COVID-19 antigen tests are faster and cheaper than the RT-PCR test, they very often produce inaccurate results. The major limitation of antigen tests is their low sensitivity, leading to high false negative outcomes, therefore it is not recommended by the WHO [6]. Moreover, it has been reported to have lower sensitivity compared to RT-PCR tests [7]. On the other hand, CT and X-ray imaging modalities play an important role in the diagnosis of lung-related abnormalities. Numerous research articles have proven the ability of the X-ray and CT modalities to achieve more accurate results than RT-PCR [8,9]. However, these imaging modalities require the presence of a skilled specialized radiologist to perform the diagnosis. Furthermore, the COVID-19 diagnosis procedure is difficult due to the symmetry among the patterns of the new coronavirus and other sorts of similar diseases [10]. Furthermore, the manual investigation requires a long time and thus automatic diagnostic tools are compulsory to decrease observation time and exertion achieved by experts to perform the diagnosis and produce a precise decision.

Artificial intelligence (AI) techniques aim to create automated diagnostic tools capable of analyzing medical data (such as images and bio-signals) simply and fast. They have been utilized successfully to enhance prognosis and diagnosis of various disorders and diseases [11,12,13,14,15,16,17,18,19,20]. The ability of AI techniques to facilitate the new coronavirus has been proven in the survey article [21]. Currently, deep learning (DL) approaches are widely used to construct automated diagnostic tools using radiograph images to support the diagnosis of COVID-19 and avoid the challenges of manual inspection [22,23]. Regardless of the achievements of DL methods in diagnosing COVID-19 using radiographic images, these scanning techniques have some limitations. These shortcomings include high cost, immobility, exposure to a large amount of radiation, and the requirement for qualified technicians to acquire these images [24]. Hence, new diagnostic tools based on other modalities are needed to assist in COVID-19 diagnosis whilst the epidemic persists.

It is well-known that COVID-19 primarily affects the respiratory system; however, it also affects the cardiovascular system [25,26]. Numerous research articles have shown various types of cardiovascular alterations in people with COVID-19. These variations involve divergence of the ST segment of the PR interval [27], arrhythmias [28], QRST changes, and conduction disorders [25]. These cardiac variations can be visualized on the electrocardiogram (ECG) of patients with COVID-19. Such cardiovascular modifications [29] have promoted the study of ECG data as a new means of diagnosing the novel coronavirus. Looking at the huge advantages of using ECG, including low cost, mobility, simplicity of use, safe, harmless, and providing real-time monitoring, automated diagnostic tools for COVID-19 based on ECG data could be of significant value in addition to imaging modalities and PCR exams. Thus, further investigation is needed to verify the feasibility of using ECG for the diagnosis

### Related Studies

The conventional method to study ECG data by AI is to mine traditional handcrafted features and employ them to train machine learning classifiers. These methods have previously been used to identify cardiac anomalies from ECG records. Numerous research articles used such methods based on 1D ECG signals to detect several cardiac problems [30,31,32,33,34]. However, these methods generally require a trade-off between accuracy and computation load and are subjective to errors [11,35]. Conversely, DL was recently employed to examine ECG by automatically attaining valuable features, thus avoiding the disadvantages of handcrafted methods [36,37]. Many studies have shown that ECG 1D signals converted to 2D demonstrations have better performance and benefits than 1D-based models [38,39]. Several studies analyzed and converted 1D to 2D ECG using transform domains such as short-term frequency transform and wavelet transform [38,39,40,41,42,43,44] and used them with DL techniques. Many studies used several forms of DL models to detect abnormalities in ECG signals [45,46,47,48,49]. It is worth mentioning the great efforts that were made by the PhysioNet/Computing in Cardiology Challenge in 2020 and 2021 to stimulate the multitype arrhythmia classification over annotated databases with thousands of 12-lead ECG recordings [50,51]. Despite the success of previous studies in detecting cardiac complications from ECG signals, it could not be easily used in real clinical practice as the above methods mainly rely on ECG signals; however, in real medical practice, this is regularly not the usual scenario. Because the ECG data taken in real clinical practice are acquired and stored as 2D ECG trace images [52]. Unlike the digital ECG signal acquired using wearable sensors, which contain multiple clean and well-detached leads, the ECG trace image data acquired in real practice are ambiguous. Such a trace image has an overlay between ECG waveforms collected from different leads and the rigid surrounding minor axes that raise hardness in mining significant information precisely. Furthermore, in digital ECG signals, that data is collected in hundreds of hertz as a sampling frequency; however, in real medical practice, the ECG data are acquired in few hertz, which results in a huge degradation in the quality of the data which correspondingly impacts the classification performance of AI-based models. One possible solution to resolve that issue is to turn the trace image into a digital ECG signal [53]. However, this conversion is complex, and the converted signal is of low quality due to the extensive noise generated by the conversion [54]. Even with the great capabilities of DL methods, this noise hinders DL techniques in detecting the small variations among different cardiac anomalies, which is the major component of cardiac complications diagnosis.

The abovementioned issues obstruct the digital ECG signals from being used in real-world clinical practice which collects ECG records as trace images. Therefore, some research articles used direct ECG trace images to identify several cardiac complications using AI techniques. The authors in [55] proposed a system to detect myocardial infarctions from ECG trace images. Their system contained multiple divisions based on shallow artificial neural networks (ANN) that used 12-lead ECG, achieving an accuracy of 94.73%. In [56], a discrete wavelet transform (DWT) was used to obtain significant features from the trace images using the ‘Haar’ wavelets. An ANN was constructed to differentiate between normal and abnormal ECG patterns, obtaining an accuracy of 99%. In [57], five hand-made feature extraction methods along with five classifiers were used to recognize two categories of cardiac arrhythmias. The highest accuracy of 96% was achieved using local binary patterns and ANN. On the other hand, Du et al. [58] proposed a DL pipeline to identify several cardiac diseases. The pipeline determined the prospective distinctive regions and adaptively merged them. Next, a recurrent neural network was employed and attained a sensitivity and precision of 83.59% and 90.42%, respectively. The MobileNet v2-deep DL method was utilized in [59] to identify four cardiac complications with 98% accuracy. In [60], DenseNet was trained with ECG trace images to predict strokes and achieved 85.82% accuracy.

The promising performance achieved using the formerly discussed methods based on ECG trace images triggered the investigation of the possibility of employing this type of ECG data with DL techniques to diagnose COVID-19. An acknowledgment must be made of the recently published public data [61] which has helped to achieve the suggested target. This data has ECG images of patients with COVID-19 and other cardiac findings. To the best of our knowledge, up to today, four studies have utilized this dataset to examine the potential of using ECG trace images in the new diagnosis of coronavirus. This dataset was used in [62] to study the impact of employing various enhancement methods on the diagnosis of COVID-19 using EfficientNet trained with ECG trace images. The paper concluded that augmentation methods are useful to some extent; nevertheless, exceeding this extent will lower the performance. An 81.8% maximum accuracy was achieved. Whereas in [63], six DL approaches were utilized to identify COVID-19 from other cardiac findings in two classification categories. Alternatively, in [64], hexaxial feature and Gray-Level Co-occurrence Matrix (GLCM) approaches were employed to extract considerable features and generate hexaxial mapping images. The created images were fed to DL methods to distinguish COVID-19 from other images as a binary classification category with a precision of 96.2%. The study [65] extracted deep features from two layers of several CNNs to an accuracy of 97.73% and 98.8% for multiclass and binary classification problems.

These previous studies experienced numerous shortcomings. Initially, the tool implemented in [64] performed only using binary classification category; however, the multiclass problem is more complicated and essential but was not considered. Furthermore, the hexaxial feature mapping utilized in it is quite sensitive to image quality, which correspondingly influences the extraction process of the GLCM procedure. The classification results obtained in the tool presented in [62] were considerably low and therefore cannot be reliable. The number of samples and features used in the testing process of the tool introduced in [63] was small, leading to a probable bias. The study [65] used a huge number of features to build their model. On the other hand, the tools proposed in [62,63,66] were based on individual DL models to perform the feature extraction or classification procedure. However, the research articles [67,68] confirmed that the incorporation of features of numerous DL approaches has the capacity to improve the classification results.

This study examined the viability of utilizing ECG information for COVID-19 diagnosis via presenting a novel diagnostic tool using various AI methods. The proposed tool attempts to overcome the limitations of the previous studies by incorporating several DL techniques and using a hybrid feature selection approach to reduce the number of features used to train the classification models. The classification procedure of the proposed tool is performed on two levels. The primary level aims to classify the ECG data to COVID-19 and normal cases (binary class level). The second level is multiclass to distinguish COVID-19 cases from normal and other cardiac complications. 

## 2. Materials and Methods

### 2.1. ECG Dataset 

The proposed diagnostic tool uses a recent dataset that is public [61], including images of ECG records for patients with COVID-19 and other cardiac problems. Until now, to the best of our knowledge, this is the primary and single public dataset for ECG records of COVID-19. ECG images available in the dataset are 1937 of distinct categories. The dataset consists of 250 scans of cases with the novel coronavirus, 300 trace records of cases with a present or former myocardial infarction (MI), 548 ECG records of irregular heartbeats, and 859 normal images without any heart complications as shown in Table 1. Data were acquired using a 12-lead system with a sampling frequency equal to 500 Hz through an EDAN SE-3 series 3-channel electrocardiograph. Table 1 also illustrates the number of images used for the training and validation sets of the proposed tool. The dimension of the images varied from 952 × 1232 to 2213 × 1572. The x-scale is 25 mm/s, and the y-scale is 10 mm/volt. Six ECG electrodes were placed on the chest representing six precordial leads. Another three electrodes were placed on the two arms and left leg representing six limb leads, including augmented voltage right (AVR), augmented voltage left (AVL), augmented voltage foot (AVF), Lead I, II, and III. The images of the dataset were evaluated by medical professionals using a telehealth ECG diagnostic scheme. This evaluation was carried out under the supervision of expert cardiologists who had long experience in ECG annotation and exploration. These medical experts removed all uncertain, ambiguous, and misleading images from the dataset.

**Figure 1:**
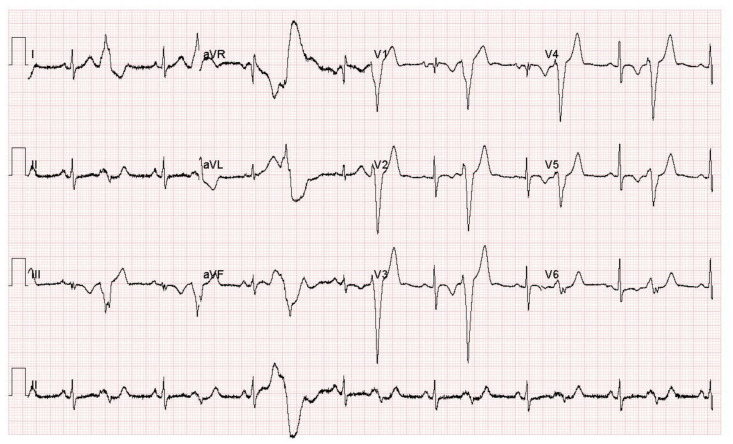
An ECG trace record sample for a COVID-19 patient.

In the binary classification level (normal versus COVID-19), 250 normal and 250 novel coronavirus records were utilized. Whereas in the multiclass classification level, a total of 750 images were employed, 250 for cardiac complications, 250 for normal cases, and 250 for COVID-19 cases. To avoid the classification bias that occurs due to the class imbalance structure of the ECG dataset (the number of images per class is not equal) that affects the classification process, an equal number of images was selected and used for each class to train the classification models. An ECG trace record sample for a COVID-19 patient is shown in Figure 1.

### 2.2. Proposed Tool

**Figure 2:**
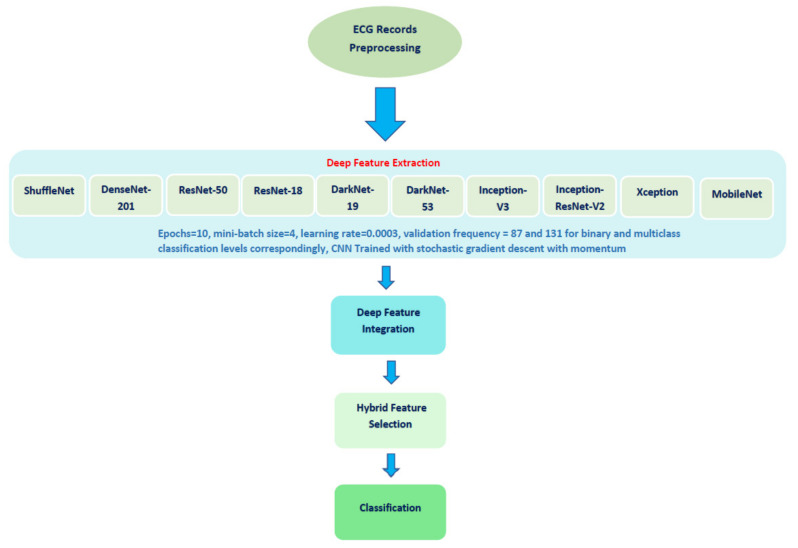
A diagram describing the steps of the proposed diagnostic tool.

The proposed automated tool consists of four steps: ECG trace image preprocessing, deep feature extraction and feature incorporation, hybrid feature selection, and classification. The proposed method used ten DL approaches. Figure 2 shows a diagram that describes the steps of the proposed diagnostic tool.

DL is an emerging technology that has been widely employed in several fields. DL approaches are the recent class of machine learning (ML). They consist of numerous architectures; however, convolution neural networks (CNNs) are the architectures most widely used for medical images [69]. Therefore, the proposed diagnostic tool utilizes ten CNNs of various architectures. These networks include InceptionResNet, ResNet-18, ResNet-50, ShuffleNet, Inception V3, MobileNet, Xception, DarkNet-19, DarkNet-53, and DenseNet-201.

**Inception V3** Google proposed the Inception CNN architecture in 2016 [70]. It is a newer version of GoogleNet [71], but with some modifications. It was first introduced to run well with reduced memory requirements and computational cost. Its principal component is the inception unit which merges numerous filters into a novel filter structure which correspondingly lowers the number of parameters. To expand the information stream into the network, the Inception module considered the depth as well as the width of the layers during the construction of the network [72].

**ResNet** is one of the time-efficient CNNs that gained popularity due to its novel structure created by He et al. in 2015 [73]. ResNet counts on the residual block which embeds crosscuts in the interior layers of a standard CNN to cross several convolution layers which quickens and eases the convergence procedure of the CNN despite the huge number of convolution layers. 

**Xception** is a new version of the Inception network introduced in 2017 [74]. The inception layers contain depthwise convolution layers, followed by a pointwise convolution layer. The Xception structure involves double layers of convolutional, then several depthwise separable convolution layers, and standard layers of convolutional and fully connected. The Xception module is more robust and powerful than the Inception module and can perform cross-channel and spatial interaction correlations while fully dissociated [75].

**Inception-ResNet-V2** presented a mixture of residual network architecture and the inception module [76]. It has a number of filters of various dimensions that are merged with residual joints. The main advantage of this fused architecture is enhancing the performance of the network and pace of convergence.

**DenseNet** was created by Huang et al. [77] in 2017, who extended the idea of shorter connections between layers near the input/output layers. The key building block of this network is the ‘dense block’. The major difference between the residual block and dense block is that the latter attaches every layer to each layer having a similar input resolution, whereas the former generates shorter links among adjacent layers. Second, each layer of DenseNet accomplishes a concatenation of the earlier outputs; in contrast, ResNet performs a summation. DenseNet-201 was utilized in this article, containing 201 layers.

**ShuffleNet** is an effective CNN primarily designed by Zhang et al. in 2018 [78]. ShuffleNet was initially produced to serve fields that require low computational capability. It contains two key blocks known as pointwise group convolution and channel shuffle. The first block utilizes convolution layers of dimension 1 × 1 to reduce training speed while attaining adequate precision. The second block supports the data flowing across feature channels by allowing a cluster of layers to control input data belonging to distinct groups, where the output/input channels are connected. 

**DarkNet** is a new DL architecture designed by the authors of [79]. It employs YOLO-V2 as the backbone of its structure. DarkNet uses filters of dimension 3 × 3 and then doubles the number of channels after every pooling phase. It employs a pooling stage to perform detection and classification as well as 1 × 1 filters to reduce the feature presentation between 3 × 3 convolutions. Darknet-19 involves 19 convolutional layers, whereas DarkNet-53 contains 53 convolutional layers.

**MobileNet** is a fine and time-efficient DL architecture that was originally designed in [80]. It can decrease the complexity of the training model by lowering the number of parameters while maintaining an acceptable performance. These are convolutional layers of dimensions 3 × 3 and 1 × 1, respectively. MobileNet has 53 deep layers.

#### 2.2.1. ECG Image Preprocessing

Initially, the dimensions of the ECG images are changed according to the input layer dimension of each CNN model. Then, those ECG records are augmented to increase the amount of records available in the data set and prevent the likelihood of overfitting that could occur in the case of small data. Those augmentation methods included in the proposed diagnostic tool are flipping in both the x- and y-orientations, and translation in both the x- and y-directions where the range of the translation distance is picked randomly within the range (−30, 30). The scaling augmentation method is also applied to the image in the x- and y-directions where the image is scaled with a scale factor chosen randomly from the range (0.9, 1.1). Table 2 demonstrates the dimensions of the input layers of each of the CNN models and the extracted features length. Table 2 shows that the number of features extracted from the last fully connected layer of each CNN for the binary classification and multiclass classification levels is 2 and 3, respectively.

#### 2.2.2. Deep Features Extraction and Feature Incorporation

Some complications may occur while CNNs are being trained, including convergence and overfitting. These issues impose the adjustment of a few parameters in the CNNs to guarantee that the weights of the CNN layers are updated at the same rate during the training process. Transfer learning (TL) is a method that can solve this problem. TL re-employs a CNN that was previously learned with a huge dataset like ImageNet for another classification problem [81]. In other words, TL uses a pretrained CNN that has learned feature representations from a large dataset to solve another classification problem dealing with a small dataset (similar to the dataset used in this paper). This process can enhance detection accuracy if used for comparable problems [81]. For that reason, this paper used ten CNNs that were pretrained. Before retraining the ten CNNs, the number of their output layers was changed to 3 or 2 which is equal to the number of classes in the case of the multiclass and binary class classification categories of the proposed diagnostic tool. In other words, the DL models were retrained for the novel classification task. Then, after the retraining process was finished, deep features were extracted from the last fully connected layers of the ten pretrained CNNs. The number of features extracted from each CNN was 2 in the case of the binary classification category and 3 in the multiclass classification category. Afterward, the proposed tool incorporated the deep features extracted from the ten DL models in a concatenation way to form one feature vector consisting of 20 and 30 features in the case of the binary and multiclass classification categories, respectively.

#### 2.2.3. Hybrid Feature Selection

Feature selection (FS) is an essential step to selecting the most valuable features available in the feature space to reduce its dimension, which correspondingly boosts the diagnostic accuracy and avoids overfitting [82,83]. FS methods can be categorized into three categories: filter, wrapper, and hybrid [84]. Hybrid FS merges filter and wrapper methods. This category combines the benefits of previous FS types [84]. Thus, a hybrid FS approach was presented and employed in this study.

The hybrid FS step presented in the diagnostic tool combines the chi-squared test filter FS approach with a wrapper FS approach based on three search strategies. The chi-squared-test is a well-known and commonly used FS method [85]. It attempts to determine the significant features *t_k_* that best differentiate positive and negative sets of instances of class *C_i_*. The chi-squared test score is calculated using Equation (1).

(1) $$\mathrm{Chi}-\mathrm{Squared}\;\mathrm{Test}=\frac{N{\left(\mathrm{AD}-\mathrm{CB}\right)}^{2}}{\left(A+C\right)\left(B+D\right)\left(A+B\right)\left(C+D\right)}$$

where N is the total number of ECG records (samples in a dataset); A = the number of samples in class *c_i_* that contain the feature *t_k_*; B = the number of samples that contain the feature *t_k_* in other classes; C = the number of samples in class *c_i_* that do not contain the feature *t_k_*; D = the number of samples that do not contain the feature *t_k_* in other classes.

The hybrid FS method initially ranks deep features extracted from the ten CNN models utilizing the chi-squared test filter FS. Then, it employs this ranking to guide the three feature search strategies within the wrapper FS approach. These three search strategies are backward, forward, and bidirectional. The first searching approach starts with all features in the feature space and then ignores features of lower ranks iteratively. Conversely, the forward approach begins with one feature having the greatest rank and then adds the following features one by one. The bidirectional alternates between the forward and backward strategies. Note that for the three strategies, only the features that improve the classification results are kept, while others are deleted.

#### 2.2.4. Classification

The classification phase was performed in two schemes. The first scheme was an end-to-end deep learning classification with ten CNNs, including InceptionResNet, ResNet-18, ResNet-50, ShuffleNet, Inception V3, MobileNet, Xception, DarkNet-19, DarkNet-53, and DenseNet-201. The second scheme used several machine learning classifiers trained with deep features extracted from the last fully connected layers of the ten CNNs. These classifiers involved a support vector machine (SVM), random forest (RF), K-nearest neighbor (KNN), the linear discriminate classifier (LDA), quadratic discriminate analysis (QDA), and decision tree (DT). The classification step included two levels: binary and multiclass. At the former level, classifiers were used to identify COVID-19 and normal patients. The multiclass level classified images into normal, COVID-19, and cardiac complications. The 10-fold cross-validation method was used to validate the results. The classifiers were run 10 times and the average classification performance of all these runs is displayed in the results section. Classification was carried out in two phases. Phase I used the deep features extracted from the ten CNN models to train the classifiers. Phase II employed the hybrid FS approach to select features used to train the classifiers.

**LDA** is a popular machine learning technique used for both classification and feature reduction. It searches for the linear combinations of features that have a high ability to explain the data. LDA separates class labels of data using hyperplanes. These planes are achieved by looking for the projection of data points that can minimize their variance and maximize the distance between class labels.

**K-NN** is a commonly used classifier in the field of machine learning due to its simplicity, straightforwardness, and effectiveness even with noisy data. Although it is simplistic, it has the ability to reach good classification accuracy in medical applications. It allocates a label to every instance in the test data equivalent to the label amongst the k nearest neighbors included in the training data. This label is chosen according to the distance measured between the instance being classified and those instances in the training data. This distance shows that instance in the test data to those in the training data. The distance used in our approach was the Euclidean similarity measure and the number of neighbors (k) was equal to 1 and 5 for binary and multiclass classification levels, respectively, with equal distance weights. 

**Decision Trees** are well-known machine learning classifiers that are widely used in medical applications due to several reasons. They are capable of visualizing interactions between extracted features. This visualization process enables a doctor to easily understand how the classifier decision is made. The DT classifier creates instances of data according to conditions. The DT has a tree structure with a root node whose leaves demonstrate class labels, and the branch nodes present the extracted features and reasons that result in this class label. The nodes of a tree are connected by an arc that represents the condition of the feature. The tree is divided into branches and leaves based on a metric such as information gain, gain ratio, or Gini index. The maximum number of splits in this study was 100, and the splitting criterion was the Gini diversity index. 

**Random Forest** is an ensemble classifier that consists of multiple decision trees. RF uses the divide-and-conquer approach (DAC) to perform classification. The DAC method divides the input feature space into several partitions depending on a goodness metric. Subsequently, the classification outputs of all trees are averaged to produce a final decision. The Gain ratio metric was used in the proposed tool. There, the number of trees was 100.

**SVM** is a robust machine learning classifier. It transforms linear or nonlinear input data points into a new domain that can easily separate between classes of data. A hyperplane is employed to separate between classes of input data to facilitate classification. A kernel function maps the similarity between the input vector and the new higher-dimension feature space. The linear kernel function was employed.

On the other hand, for retraining the CNNs for end-to-end classification, the learning rate, number of epochs, and minimum batch size were adjusted to 0.0003, 10, and 4, respectively. Whereas the validation frequency was modified to 87 and 131 for binary and multiclass classification levels, respectively. The ten CNNs were trained with the stochastic gradient descent with a momentum algorithm. The other hyperparameters were kept unchanged. The proposed diagnostic tool was implemented using the Weka Data Mining Tool [86] and MATLAB R2020a.

### 2.3. Performance Evaluation 

The overall performance of the proposed diagnostic tool was measured using multiple metrics involving the Mathew correlation coefficient (MCC), the F1 score, precision, specificity, and sensitivity calculated using Equations (2)–(7). In addition to confusion, the receiver operating characteristics curve (ROC) and the area under ROC (AUC) were also determined.

(2) $$\mathrm{Accuracy}=\frac{\mathrm{TP}+\mathrm{TN}}{\mathrm{TN}+\mathrm{FP}+\mathrm{FN}+\mathrm{TP}}$$

(3) $$\mathrm{Sensitivity}=\frac{\mathrm{TP}}{\mathrm{TP}+\mathrm{FN}}$$

(4) $$\mathrm{Precision}=\frac{\mathrm{TP}}{\mathrm{TP}+\mathrm{FP}}$$

(5) $$\mathrm{MCC}=\frac{\mathrm{TP}\times \mathrm{TN}-\mathrm{FP}\times \mathrm{FN}}{\sqrt{\left(\mathrm{TP}+\mathrm{FP}\right)\left(\mathrm{TP}+\mathrm{FN}\right)\left(\mathrm{TN}+\mathrm{FP}\right)\left(\mathrm{TN}+\mathrm{FN}\right)}}$$

(6) $$F1-\mathrm{Score}=\frac{2\times \mathrm{TP}}{\left(2\times \mathrm{TP}\right)+\mathrm{FP}+\mathrm{FN}}$$

(7) $$\mathrm{Specificity}=\frac{\mathrm{TN}}{\mathrm{TN}+\mathrm{FP}}$$

where FN refers to the false negative which is the amount of COVID-19 records wrongly categorized as nonCOVID-19, TN is the true negative representing the nonCOVID-19 records correctly recognized. TP is the true positive, which is equal to the number of COVID-19 scans properly identified. Finally, FP is the false positive equivalent to the sum of nonCOVID-19 records improperly classified as COVID-19.

## 3. Results

### 3.1. Phase I Classification Results

**Figure 3:**
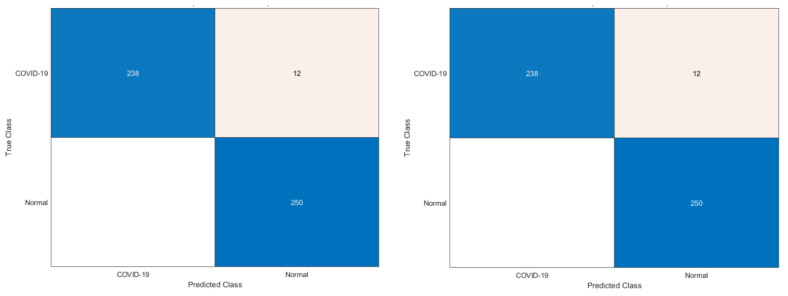
Confusion matrices for the binary class classification level: (**left**) LDA, (**right**) SVM classifiers.

**Figure 4:**
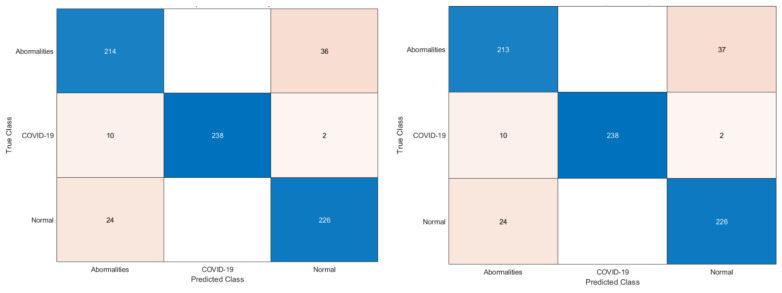
Confusion matrices for the multiclass classification level: (**left**) LDA, (**right**) SVM classifiers.

**Figure 5:**
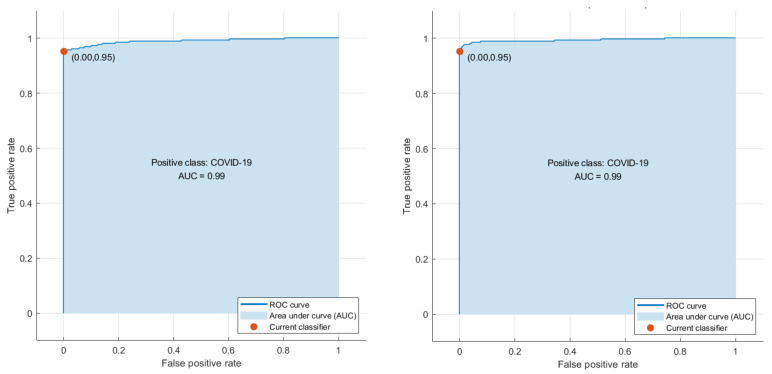
The ROC curves for the binary class classification level: (**left**) LDA, (**right**) SVM classifiers.

**Figure 6:**
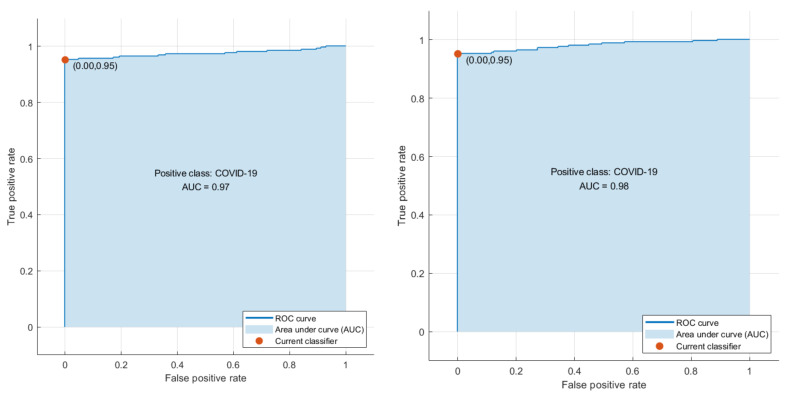
The ROC curves for the multiclass classification level: (**left**) LDA, (**right**) SVM classifiers.

**Figure 7:**
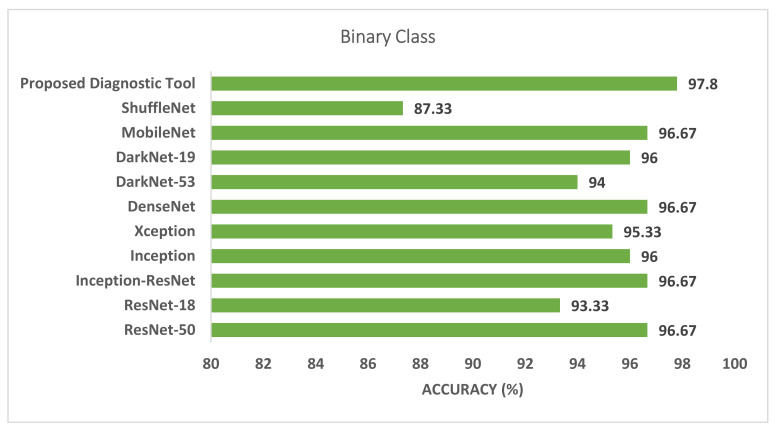
The classification accuracy of the proposed diagnostic tool using the RF classifier of phase I compared to the end-to-end DL classification for the binary level.

Phase I represents the use of deep features extracted from the ten CNNs and fused to train the machine learning classifiers. Table 3 illustrates the classification accuracy of phase I for the binary class and multiclass classification levels, respectively. Table 3 shows that the maximum accuracy of 97.78% was achieved for the binary classification level using the RF model. All other classifiers obtained an accuracy that ranged from 97.36% to 97.6%. The highest accuracy of 90.88% was achieved using the RF classifier for multiclass classification. The SVM, LDA, and KNN achieved the next-highest accuracies of 90.43%, 90.35%, and 89.39%. Finally, the DT and QDA classifiers reached the lowest accuracies of 86.56% and 85.6%, respectively. The confusion matrices attained using the LDA and SVM classifiers are shown in Figure 3 and Figure 4 for binary and multiclass classification, respectively. The ROC curve for the SVM and LDA classifiers are shown in Figure 5 and Figure 6 for binary and multiclass, respectively. Figure 7 shows that the AUC for the LDA and SVM classifiers were 0.99 and 0.99 for the binary class classification level. For the multiclass classification level, the AUCs for the LDA and SVM classifiers were 0.97 and 0.98, respectively.

The two Figures S1 and S2 have been attached to the Supplementary Materials representing a two-dimensional scatter plot of the first two features of the feature space for the binary and multiclass classification levels used as inputs to the classifiers. Also, the two Figures S3 and S4 have been added to the Supplementary Materials representing a two-dimensional scatter plot of the LDA classifier predictions using the first two features of the feature space for the binary and multiclass classification levels. Moreover, the two Figures S5 and S6 have been added to the Supplementary Materials representing a two-dimensional scatter plot of the LDA classifier predictions using the first two features of the feature space for the binary and multiclass classification levels

To access and confirm the statistical significance of the performance of the ML classifiers, the one-way analysis of variance (ANOVA) test was applied to the results of the classifiers after a repeated 10-fold cross-validation process. The ANOVA test was performed on the classification accuracy results achieved using the classifiers of the binary classification level to test the statistical significance between them. The results are shown in Table 4. ANOVA was also performed for the results of the multiclass classification problem and the outputs of the test are shown in Table 5. It can be seen in Table 4 and Table 5 that the p-values attained from the test were lower than α, where α = 0.05. Consequently, it could be concluded that there is a statistically significant difference in the classification accuracies of the classifiers for both the multiclass and binary classification levels.

**Figure 8:**
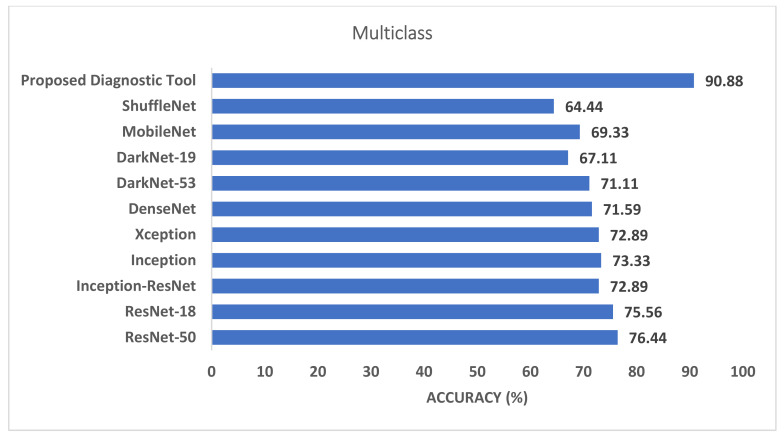
The classification accuracy of the proposed diagnostic tool using the phase I RF classifier compared to the end-to-end DL classification for the multiclass level.

Figure 7 and Figure 8 compare the phase I performance of the RF classifier of the proposed diagnostic tool with the end-to-end DL classification for the binary and multiclass levels. Figure 7 proves that the deep features extracted from the last fully connected layers of the ten CNNs had a higher classification accuracy compared to end-to-end pretrained CNNs for the binary classification level. On the other hand, for the multiclass classification level, the RF classifier of the proposed diagnostic tool obtained 90.88% accuracy, which is higher than all other pretrained CNNs. As can be seen in Figure 8, the accuracy of the RF classifier of the proposed diagnostic tool was greater than the 76.44%, 75.56%, 72.89%, 73.33%, 72.89%, 71.59%, 71.11%, 67.11%, 69.33%, and 64.44% achieved using ResNet-50, ResNet-18, Inception-ResNet, Inception, Xception, DenseNet-201, DarkNet-53, DarkNet-19, MobileNet, and ShuffeNet, respectively.

### 3.2. Phase II Classification Results

Phase II of the proposed diagnostic tool presented the features selected after the hybrid FS approach used them to train the classification models. The following section presents the results of the hybrid feature selection approach based on the three search strategies using three classifiers. First, it shows the rank scores of features using the chi-square test filter FS method. Then, it shows the number of selected features as well as the classification accuracy attained for the binary and multiclass classification levels. Table 6 and Table 7 represent the ranking score for each feature attained using the chi-square test FS method for the binary and multiclass classification levels, respectively.

Table 8 shows the binary class classification level after the hybrid FS approach of the proposed diagnostic tool using the three search strategies (phase II) compared to phase I (before FS) for the DT, RF, and QDA classifiers as they achieved the highest accuracies in phase I. Table 8 shows that the hybrid FS approach of the proposed diagnostic tool improved the classification accuracy compared to phase I. This was obvious as the accuracies attained using the forward and bidirectional strategies were 98.2%, 98%, and 97.8%, which are better than those attained before FS. In addition, the accuracies attained using the backward strategy were 98.2%, 98%, 97.6% for the DT, RF, and QDA classifiers, which were higher than those achieved before FS using the same classifier except for the QDA which is equal to that achieved before FS. Some performance measures were calculated for the binary classification level and are illustrated in Table 9. Table 9 reveals the results for the sensitivity (0.968, 0.96, 0.956), specificity (0.996, 1, 1), precision (0.996, 1, 1), F1-score (0.982, 0.961, 0.978), and MCC (0.964, 0.989, 0.957) for the DT, RF, and QDA models, respectively, using the forward search strategy. 

On the other hand, the results of the multiclass classification level of phase II of the proposed diagnostic tool are displayed in Table 10 and Table 11. Table 10 shows the multiclass accuracy of the hybrid FS approach of the proposed diagnostic tool using the three search strategies (phase II) compared to phase I (before FS) for the RF, LDA, and SVM classifiers which achieved the highest accuracy in phase I. The accuracies displayed in Table 10 verify that the hybrid FS approach based on the three search methods increased the capacity of the classification model compared to phase I (before FS). This was clear as the forward and bidirectional strategies achieved better accuracies of 91.6% and 90.93% for the RF classifiers, 91.07% and 91.33 for the LDA classifier, and 90.58% and 90.53% for the SVM classifier compared to 90.88%. Using the exact classifiers before FS, 90.35% and 90.43% accuracy was achieved. Similarly, the backward search method reached accuracies of 91.33%, 91.07%, and 90% using the RF, LDA, and SVM classifiers, which were higher than those attained before FS except for the SVM classifier, it remained the same. Table 11 indicates the sensitivity (0.916, 0.911, 0.905), specificity (0.958, 0.955, 0.953), precision (0.918, 0.918, 0.908), F1 score (0.917, 0.917, 0.906), and MCC (0.875, 0.875, 0.859) for the RF, LDA and SVM classifiers, respectively, using the forward search method. 

**Figure 9:**
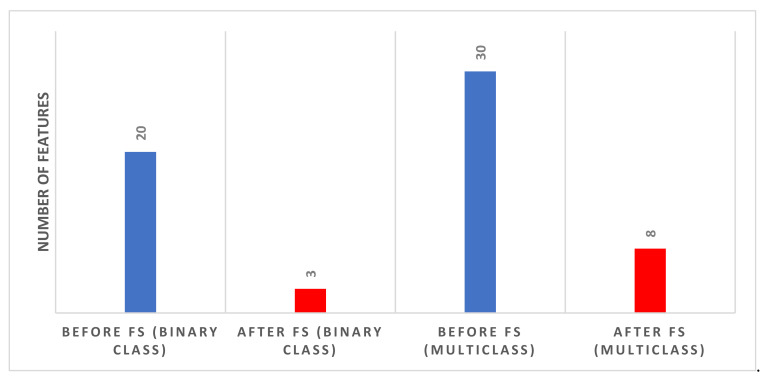
The number of features of phase I and phase II for the binary and multiclass classification levels using forward search strategies (using the classifiers which attained the peak performance).

Figure 9 shows the number of features selected for the binary and multiclass levels using the forward search strategy that reached maximum accuracy (using the DT classifier for binary and the RF model for multiclass). Figure 9 indicates that the number of features after the hybrid FS for the binary classification problem is three. The three features include Feature 2 of MobileNet, Feature 1 of InceptionResNet, and Feature 2 of ResNet-50. The figure also shows that the number of features after FS for the multiclass classification level is eight. These eight features are Feature 1 of MobileNet, Feature 3 of Inception, Feature 3 of ResNet-18, Feature 1 of Xception, Feature 2 of DarkNet-53, Feature 3 of DarkNet-53, Feature 1 of DarkNet-19, and Feature 3 of InceptionResNet.

## 4. Discussion

Recent relevant studies revealed various forms of cardiovascular variations in ECG data acquired from patients infected by the novel coronavirus as ST-segment changes, QRST irregularities, and arrhythmias. On the other hand, several research articles discussed that COVID-19 could not be the leading reason for these cardiovascular deformities; nevertheless, it should be emphasized that it could reveal the intrinsic conditions or lower them [87]. The entire cardiac findings indicated in the literature have been observed on all the ECG data utilized in this study. 

This paper presented a novel diagnostic tool to automatically diagnose COVID-19 by incorporating multiple DL and hybrid FS approaches. This diagnostic tool consists of two classification levels: binary and multiclass. The first level consists of distinguishing COVID-19 and normal cases, while the second level consists of recognizing COVID-19, normal, and other cardiac abnormalities. The proposed tool extracted deep features from the last fully connected layers of ten CNNs models. Next, it fused these features, used several classifiers in the two classification levels, and compared their performance with the end-to-end DL classification. The previous step is known as phase I of the proposed diagnostic tool. Afterward, a hybrid FS method was presented based on three search approaches. This process is called phase II of the proposed diagnostic tool. The results achieved in phase I showed that the deep feature incorporation is better than end-to-end DL classification as shown in Figure 7 and Figure 8. Phase I of the proposed tool attained an accuracy of 97.78% and 90.88% for the binary and multiclass classification levels, respectively. These accuracies are greater than those obtained by the end-to-end deep learning classification, having a range of 87.33–96.67% and 64.44–76.44% for the binary and multiclass classification levels, respectively.

In the second phase of the proposed tool, only classifiers that attained the highest accuracies for either the binary or the multiclass classification levels were employed in the hybrid FS procedure. Table 8 compares the results before and after feature selection for the three classifiers which attained the highest accuracies for the binary classification level. Table 8 shows that the highest accuracy of 98.2% was achieved using DT trained with only three features selected during the FS process of the binary classification level. This accuracy is greater than the 97.62% achieved before FS using the same classifier trained with 20 features. Similarly, Table 10 compares the results before and after FS for the three classifiers which attained the highest accuracies for the multiclass classification level. Table 10 indicates that the maximum accuracy of 91.6% was reached using the RF classifier trained with only eight features chosen during the FS procedure of the multiclass classification level. This accuracy is greater than the 90.56% accomplished before FS using the same classifier learned with 30 features. Thus, the performance of phase II of the proposed tool verifies that the presented hybrid FS method had a further enhancement in classification performance. It also reduced the number of features successfully. 

It is worth mentioning that ECG detection requires more physical contact between patients and physicians than the RT-PCR test or CT imaging, which will increase the risk of virus transmission. Therefore, ECG may be more suitable as an auxiliary inspection means of COVID-19 than a primary screening tool.

### 4.1. Comparison with Related Studies

The performance of phase II of the proposed diagnostic tool versus other relevant tools that are directly copied from published papers is demonstrated in Table 12. The ECG records used to construct the proposed diagnostic tool are added to Appendix A. The results illustrated in Table 12 show that the proposed tool could be used to distinguish between normal and COVID-19 cases. It can also differentiate between normal, COVID-19, and other cardiac abnormalities. The table also indicates that the proposed tool has a performance comparable to those of other related studies. It is worth mentioning that, for the binary classification level, the specificity and precision of the proposed tool are higher than in the other studies [63,64]. However, it has lower sensitivity than other studies. However, for the multiclass classification level, the proposed tool achieved higher sensitivity, specificity, and precision than the studies [62,63]. These results indicate that the proposed tool based on ECG data could be used to diagnose COVID-19. It could be considered a possible novel solution that might be utilized in actual medical scenarios. It can be considered an alternative to current diagnostic tools.

### 4.2. Limitations

This study has several limitations. The first limitation is the small database for training/validation, which is quite insufficient for deep learning of thousands of hyperparameters. In addition, the lack of an independent test dataset is considered another limitation. Furthermore, this study did not consider methods that handle the class imbalance problem. Furthermore, the baseline rhythm of each patient is not available, and the effect of the baseline rhythm is not explored. Additionally, this study did not take into account optimization techniques for the selection of deep learning hyperparameters. In addition, the dataset used in the study is from confirmed COVID-19 patients. The detection of asymptomatic infections may not achieve the same level of sensitivity. Thus, the extension of the scope of the results is to some extent limited. Finally, this study did not consider the uncertainty of the input data.

## 5. Conclusions

The current study explored the prospect of employing ECG trace images for diagnosing the novel coronavirus. It proposed a novel automated ECG-based diagnostic tool that incorporates deep features from ten DL models. The proposed diagnostic tool used several well-known ML classifiers for classification. The classification procedure was performed on two levels. The primary level aimed to distinguish patients with COVID-19 from normal cases (binary class level). Whereas the second level was multiclass to distinguish cases of COVID-19 from normal and other cardiac complications. The major contributions of the diagnostic tool were, first, the construction of a novel automatic, inexpensive, harmless, susceptible, and quick diagnostic tool as a replacement to the present diagnostic tools to support the automatic detection of COVID-19. In addition, the novel tool relied on 2D ECG trace images to diagnose COVID-19, which is a new approach to achieving a diagnosis. Moreover, in view of the disparities in the performances between DL models, the proposed tool utilized ten DL models of distinctive structures to merge their benefits, not a single architecture. Additionally, it extracted features from the last fully connected layers of the ten DL models instead of end-to-end DL classification (as in previous studies). The proposed tool merged these features to investigate the impact of merging on diagnostic accuracy. Furthermore, it presented a hybrid FS approach based on three search strategies to select the most significant deep features and the lower dimensions of the feature space. Finally, it explored whether the hybrid FS approach boosts the performance of the proposed diagnostic tool. The results achieved using the proposed tool could be evidence that ECG records can be used in diagnosing the new coronavirus. The presented tool may prevent the shortcomings of chest imaging techniques, antigen, and PCR exams. It could be considered an easy, inexpensive, quick, portable, and sensible approach. Therefore, it might help clinicians in diagnosing COVID-19 accurately and automatically. Upcoming experiments will test the efficiency of the proposed tool in actual clinical procedures. Further work will consider using resampling techniques that handle the class imbalance problem. Future work will explore more deep learning techniques as well as hyperparameter optimization approaches. In addition, the uncertainty of the input data will be taken into consideration in future work.

## Figures and Tables

**Table 1:** Description of the ECG dataset including the number of available ECG images per class and the number of images used in the proposed study.

Class	Number of Available Images	Images Used in the Proposed Study	Images Used in Training	Images Used in Validation
COVID-19	250	250	175	75
Normal	859	250	175	75
Cardiac Abnormalities include:	848	250	175	75
Irregular Heartbeats	548	125	88	37
Current and Recovered MI	300	125	88	37

**Table 2:** The dimensions of the input layers of each of the CNN models and the mined features dimensions.

CNN Construction	Dimension of Input	Length of the Extracted Deep Features
**ResNet-50** **ResNet-18** **DenseNet-201** **ShuffleNet** **MobileNet**	224 × 224 × 3	Binary Classification Level
		2
		Multiclass Classification Level
		3
**Inception-V3** **Inception-ResNet** **Xception**	229 × 229 × 3	Binary Classification Level
		2
		Multiclass Classification Level
		3
**DarkNet-19** **DarkNet-53**	226 × 226 × 3	Binary Classification Level
		2
		Multiclass
		3

**Table 3:** Phase I classification accuracy (%) and standard deviations obtained using machine learning classifiers.

Binary Classification Level					
DT	RF	QDA	LDA	SVM	KNN
97.62 (0.14)	97.78 (0.06)	97.6 (0)	97.6 (0)	97.6 (0)	97.36 (0.23)
**Multiclass classification Level**					
**DT**	**RF**	**QDA**	**LDA**	**SVM**	**KNN**
86.56 (0.8)	90.88 (0.19)	85.6 (0.06)	90.35 (0.21)	90.43 (0.28)	89.39 (0.30)

**Table 4:** One-way analysis of variance test details for the binary classification level.

Source of Variation	SS	df	MS	F	*p* Value
Columns	0.901	5	0.180	12.54	<0.001
Error	0.776	54	0.014		
Total	1.677	59			

**Table 5:** One-way analysis of variance test details for the multiclass classification level.

Source of Variation	SS	df	MS	F	*p* Value
Columns	252.148	5	50.429	298.07	<0.001
Error	9.136	54	0.1692		
Total	261.284	59			

**Table 6:** The ranking score for each feature attained using chi-square FS along with its order in the feature vector and the name of the feature for the binary classification level.

Rank	Order	Feature Name
461.538	18	Feature 2 of MobileNet
461.538	5	Feature 1 of InceptionResNet
460.68	6	Feature 2 of InceptionResNet
457.854	17	Feature 1 of MobileNet
457.854	7	Feature 1 of Xception
457.592	13	Feature 1 of DarkNet-53
456.608	14	Feature 2 of DarkNet-53
456.397	19	Feature 1 of Shuffle
454.198	3	Feature 1 of Inception
454.198	2	Feature 2 of ResNet-50
454.198	4	Feature 2 of Inception
454.198	20	Feature 2 of Shuffle
454.198	10	Feature 2 of DenseNet
454.198	8	Feature 2 of Xception
454.198	15	Feature 1 of DarkNet-19
454.198	16	Feature 2 of DarkNet-19
454.198	12	Feature 2 of ResNet-18
454.198	9	Feature 1 of DenseNet
454.198	11	Feature 1 of ResNet-18
454.198	1	Feature 1 of ResNet-50

**Table 7:** The ranking score for each feature attained using chi-square FS along with its order in the feature vector and the name of the feature for the multiclass classification level.

**Rank**	**Order**	**Feature Name**
1021.0997	1	Feature 1 of ResNet-50
980.0815	25	Feature 1 of MobileNet
938.3733	19	Feature 1 of DarkNet-53
932.5696	12	Feature 3 of Xception
926.9128	28	Feature 2 of Shuffle
917.393	6	Feature 3 of Inception
916.3032	18	Feature 3 of ResNet-18
906.3512	13	Feature 1 of DenseNet
898.5766	10	Feature 1 of Xception
894.4025	15	Feature 3 of DenseNet
886.2739	3	Feature 3 of ResNet-50
883.1262	7	Feature 1 of InceptionResNet
877.7686	21	Feature 3 of DarkNet-53
865.6989	22	Feature 1 of DarkNet-19
814.21	2	Feature 2 of ResNet-50
811.4717	24	Feature 3 of DarkNet-53
798.1348	16	Feature 1 of ResNet-18
797.0761	27	Feature 3 of MobileNet
781.2226	4	Feature 1 of Inception
760.9445	8	Feature 2 of InceptionResNet
755.4454	29	Feature 2 of Shuffle
723.6848	11	Feature 2 of Xception
720.4829	23	Feature 1 of DarkNet-19
703.0506	5	Feature 2 of Inception
697.2656	20	Feature 2 of DarkNet-53
697.2656	26	Feature 2 of MobileNet
697.2656	17	Feature 2 of ResNet-18
697.2656	14	Feature 2 of DenseNet
673.7605	9	Feature 3 of InceptionResNet
634.5971	30	Feature 3 of Shuffle

**Table 8:** The accuracy of binary-level classification (%) of the DT, RF, and QDA classifiers that obtained the highest accuracy in phase I compared to after using the three search strategies of the hybrid FS approach (phase II of the proposed diagnostic tool).

Classifier	Before FS	Forward	Backward	Bidirectional
**DT**	97.62	98.2	98.2	98.2
**RF**	97.78	98.0	98.0	98.0
**QDA**	97.6	97.8	97.6	97.8

**Table 9:** The binary-level performance metrics (%) of the DT, RF, and QDA classifiers that achieved the highest accuracy using the forward search strategies of the hybrid FS approach.

Classifier	Sensitivity	Specificity	Precision	F1-Score	MCC
**DT**	96.8	99.6	99.6	98.2	96.4
**RF**	96.0	100	100	96.1	98.9
**QDA**	95.6	100	100	97.8	95.7

**Table 10:** The classification accuracy (%) of the RF, LDA, and SVM that obtained the highest accuracy in phase I compared to after using the three search strategies of the hybrid FS approach (phase II of the proposed diagnostic tool).

Classifier	Before FS	Forward	Backward	Bidirectional
**RF**	90.88	91.6	91.33	90.93
**LDA**	90.35	91.07	91.07	91.33
**SVM**	90.43	90.58	90	90.53

**Table 11:** The multiclass-level performance metrics (%) of the RF, LDA, and SVM that achieved the highest accuracy using the forward search strategies of the hybrid FS approach.

Classifier	Sensitivity	Specificity	Precision	F1-score	MCC
**RF**	91.6	95.8	91.8	91.7	87.5
**LDA**	91.1	95.5	91.8	91.7	87.5
**SVM**	90.5	95.3	90.8	90.6	85.9

**Table 12:** The results of phase II of the proposed diagnostic tool versus other related studies that are directly copied from published papers.

Binary Classification Level					
Article	Technique	Sensitivity (%)	Precision (%)	Specificity (%)	Accuracy (%)
[64]	hexaxial feature mapping + GLCM + CNN	98.4	94.3	94	96.2
[63]	ResNet-18	98.6	98.5	96	98.62
**Presented diagnostic tool**	Fully connected deep features + hybrid FS (forward search with DT classifier)	96.8	99.6	99.6	98.2%
**Multiclass Classification Level**					
		**Sensitivity (%)**	**Precision (%)**	**Specificity (%)**	**Accuracy (%)**
[62]	EfficientNet	75.8	80.8	-	81.8
[63]	MobileNet	90.8	91.3	92.8	90.79
**Presented diagnostic tool**	Fully connected deep features + hybrid FS (forward search with RF classifier)	91.6	91.8	95.8	91.6

## Data Availability

The dataset employed in this paper can be found in the Mendeley [https://data.mendeley.com/datasets/gwbz3fsgp8/2]. (accessed on 1 September 2021).

## Supplementary Materials

The following supporting information can be downloaded at: https://www.mdpi.com/article/10.3390/bios12050299/s1, Figure S1: a two-dimensional scatter plot of the first two features of the feature space for the binary classification level; Figure S2: a two-dimensional scatter plot of the first two features of the feature space for the multiclass classification level; Figure S3: a two-dimensional scatter plot of the LDA prediction using the first two features of the feature space for the binary classification level; Figure S4: a two-dimensional scatter plot of the LDA prediction using the first two features of the feature space for the multiclass classification level. Figure S5: a two-dimensional scatter plot of the QDA prediction using the first two features of the feature space for the binary classification level; Figure S6: a two-dimensional scatter plot of the QDA prediction using the first two features of the feature space for the multiclass classification level.

## Abbreviations

AI	Artificial intelligence
ANOVA	Analysis of variance
ANN	Artificial neural networks
AVF	Augmented voltage foot
AVL	Augmented voltage left
AVR	Augmented voltage right
CNN	Convolutional Neural Network
CT	Computed Temography
DL	Deep learning
DT	Decision Tree
DWT	Discrete wavelet transform
ECG	Electrocardiogram
FN	False negative
FP	False positive
FS	Feature Selection
GLCM	Gray-Level Co-Occurrence Matrix
KNN	K-nearest neighbor
LDA	Linear discriminate analysis
MI	Myocardial infarction
ML	Machine learning
QDA	Quadratic discriminate analysis
RF	Random Forest
RT-PCR	Real-time reverse transcription-polymerase chain reaction
SVM	Support vector machine
TL	Transfer learning
TN	True negative
TR	True positive
TML	Traditional machine learning techniques
WHO	World Health Organization

## Appendix A

The patients’ records that have been used are as follows:

For the normal class: records utilized are normal (1) to normal (250).

For the COVID-19 class: all records available in the dataset are utilized

For cardiac abnormalities class: abnormal heartbeats(HB) records utilized are HB(1) to HB (88), myocardial infarction (MI) records used are MB (1) to MB (77), previous myocardial infarction (PMI) records used are PMI (1) to PMI (88).
