# Supplementary material for: An Intelligent ECG-Based Tool for Diagnosing COVID-19 via Ensemble Deep Learning Techniques
Source: Biosensors (Basel). 2022 May 5;12(5):299. doi: 10.3390/bios12050299 (PMC9138764; doi:10.3390/bios12050299)
Supplement: Supplementary file 1 [file biosensors-12-00299-s001.zip › biosensors-1649886 supplementary.pdf]

biosensors

*Article*

# An Intelligent ECG-Based Tool for Diagnosing COVID-19 via Ensemble Deep Learning Techniques

**Omneya Attallah**

Department of Electronics and Communications Engineering, College of Engineering and Technology, Arab Academy for Science, Technology and Maritime Transport, Alexandria 1029, Egypt; o.attallah@aast.edu

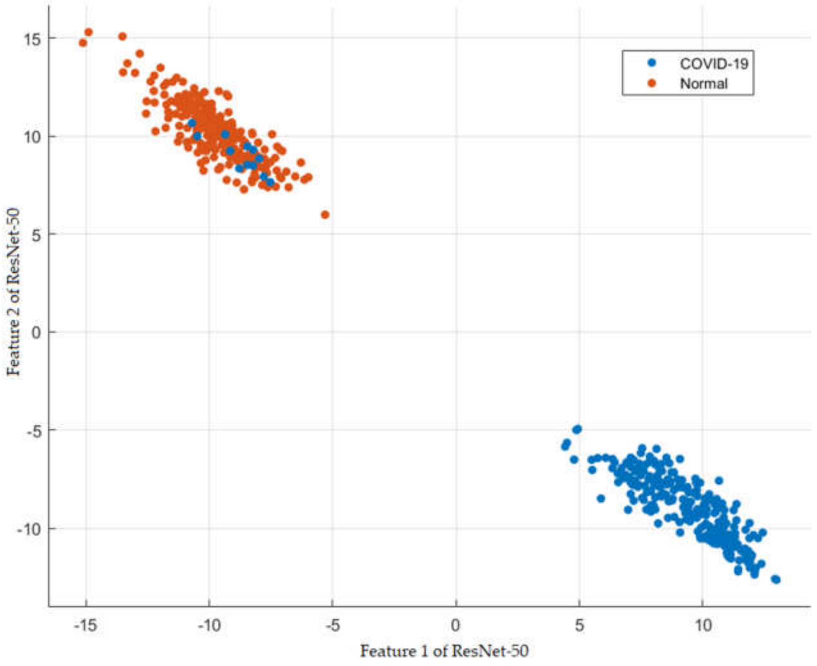


**Figure S1.** a two-dimensional scatter plot of the first two features of the feature space for the binary classification level.

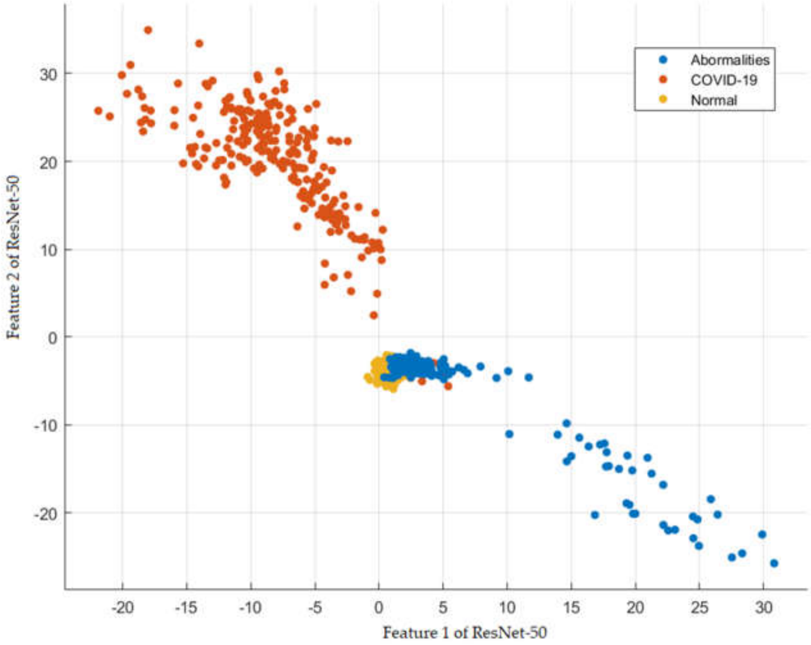


**Figure S2.** a two-dimensional scatter plot of the first two features of the feature space for the multiclass classification level.

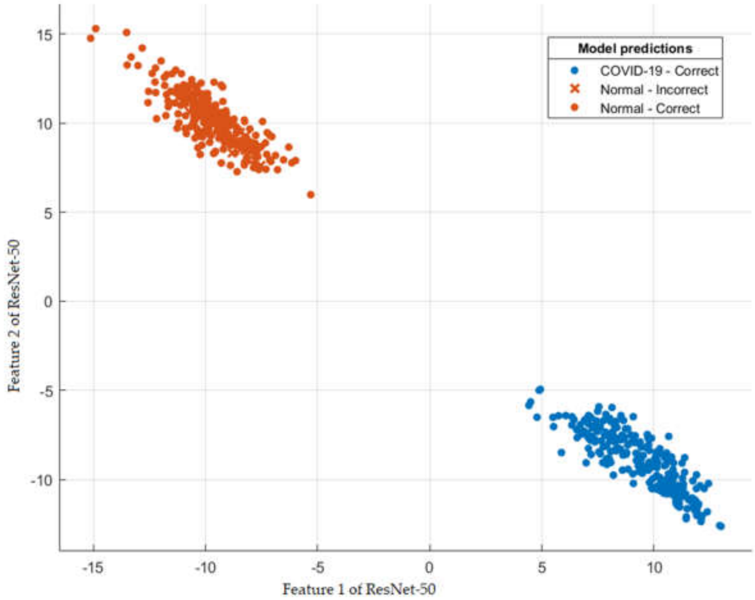


**Figure S3.** a two-dimensional scatter plot of the LDA prediction using the first two features of the feature space for the binary classification level.

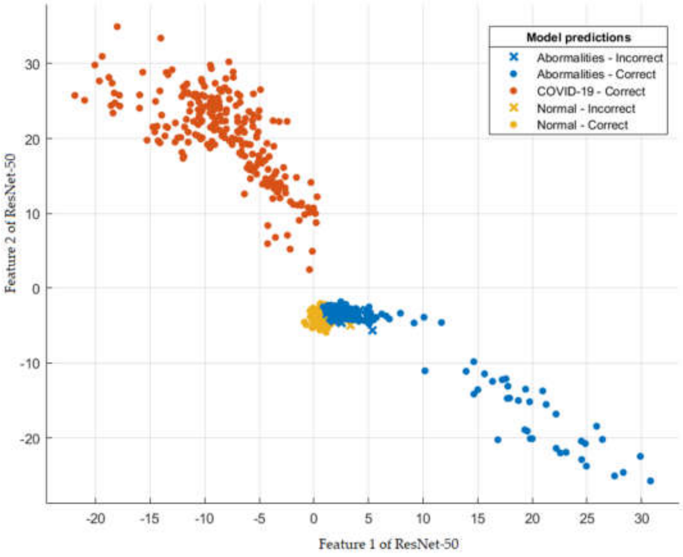


**Figure S4.** a two-dimensional scatter plot of the LDA prediction using the first two features of the feature space for the multiclass classification level.

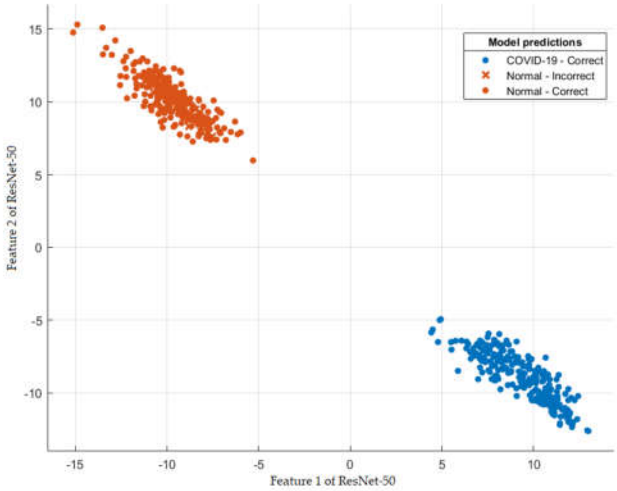


**Figure S5.** title; a two-dimensional scatter plot of the QDA prediction using the first two features of the feature space for the binary classification level.

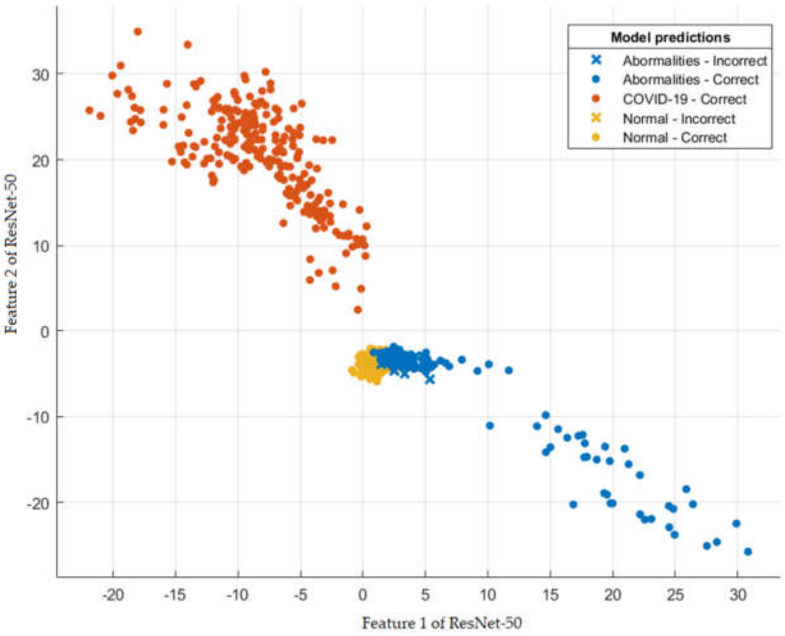


**Figure S6.** a two-dimensional scatter plot of the QDA prediction using the first two features of the feature space for the multiclass classification level.
